# Simulation-Based Preparation for the American Board of Emergency Medicine Certifying Exam: A Comprehensive Curriculum for Residents

**DOI:** 10.21980/J8.53814

**Published:** 2025-12-31

**Authors:** Kimberly Sokol, Alaa Aldalati, Michelle Hughes, Stephanie Stapleton, Charles Lei

**Affiliations:** * Kaweah Health Medical Center, Department of Emergency Medicine, Visalia, CA; ^ University of Kansas School of Medicine-Wichita, Wesley Medical Center Emergency Department, Wichita, KS; † University of Wisconsin School of Medicine and Public Health, Department of Emergency Medicine, Madison, WI; ** Boston University/Boston Medical Center, Department of Emergency Medicine, Boston, MA; ^^ Hennepin County Medical Center, Department of Emergency Medicine, Minneapolis, MN

## Abstract

**Audience and type of curriculum:**

This curriculum is designed for junior and senior emergency medicine (EM) residents who are preparing for the new American Board of Emergency Medicine (ABEM) Certifying Exam.

**Length of curriculum:**

This curriculum can be completed over the course of an EM residency program to prepare junior residents for the individual content areas encountered on the new Certifying Exam. Alternatively, it can be implemented during a single session to simulate the actual exam for senior residents.

**Introduction:**

With ABEM transitioning to a new format for its Certifying Exam, there is a critical need for targeted preparatory materials that reflect these changes.

**Educational Goals:**

The goal of this curriculum is to equip residents with the knowledge and skills needed to succeed on the ABEM Certifying Exam. It includes a comprehensive set of case types expected to appear on the Certifying Exam, with a focus on assessing competencies not currently evaluated by the existing written Qualifying Exam and retiring Oral Exam. The curriculum is designed to be delivered to current residents in a single-day exam format to closely replicate the structure and experience of the new Certifying Exam.

**Educational Methods:**

The educational strategy used in this curriculum consists of a set of eight simulation scenarios written in an Observed Structured Clinical Examination (OSCE) format. Each scenario targets a distinct area of the recently introduced ABEM Certifying Exam. These content areas include clinical decision-making, prioritization, reassessment, difficult conversations, managing conflict, ultrasound, procedural skills, and patient-centered communications. The OSCE structure intentionally reflects that of the ABEM Certifying Exam to enhance realism, ensure consistency, and maintain educational relevance.

**Research Methods:**

This eight-case simulation curriculum focuses on core EM competencies, including decision-making, communication, conflict resolution, prioritization, procedural skills, and ultrasound. Initially developed by experts in simulation and medical education, each case was subsequently refined through a structured peer review process. This process involved written evaluations by external reviewers followed by pilot testing across multiple EM residency programs and at the Society for Academic Emergency Medicine Annual Meeting. Faculty facilitators and resident learners provided targeted feedback using the Simulation Scenario Evaluation Tool^1^ and modified usability surveys, assessing factors such as case realism, scenario flow, clarity of learning objectives, alignment of assessment criteria, and practical feasibility for implementation.

**Results:**

Pilot testing across multiple institutions and at a national academic meeting demonstrated the curriculum’s strong educational value, clarity, and usability. Feedback from both facilitators and residents was overwhelmingly positive, highlighting the simulation scenarios’ realism, clinical relevance, and effectiveness in exam preparation.

**Discussion:**

This comprehensive simulation-based curriculum, designed to align with the ABEM Certifying Exam, proved to be feasible, effective, and well-received by both learners and facilitators. Key insights from its implementation emphasized the importance of thorough faculty preparation, flexibility in adapting to available resources, and the use of structured debriefing to support learning. Thoughtfully designed simulation experiences can significantly enhance EM resident preparedness for high-stakes assessments.

**Topics:**

Certifying Exam, simulation, board certification, American Board of Emergency Medicine, residency.

## USER GUIDE

**Table:** 

List of Resources:	
Abstract	1
User Guide	3
Didactics and Hands on Curriculum Chart	7
Overview of the American Board of Emergency Medicine Certifying Exam	15

**Learner Audience:**

This curriculum is designed for interns and junior and senior residents in preparation for the ABEM Certifying Exam.

**Length of Curriculum:**

This curriculum can be delivered longitudinally over the course of an emergency medicine (EM) residency to prepare junior residents for the specific content areas encountered on the new American Board of Emergency Medicine (ABEM) Certifying Exam. Alternatively, it can be administered during a single session to simulate the full exam for senior residents.

**Topics:**

Certifying Exam, simulation, board certification, American Board of Emergency Medicine, residency.

**Objectives:**

Upon completion of the curriculum, learners should be able to:

1. List the eight content categories of the ABEM Certifying Exam and describe the overall exam structure
2. Summarize the key clinical concepts and examination strategies for each of the eight content categories of the ABEM Certifying Exam
3. Apply clinical decision-making, ultrasound, and procedural skills by successfully completing all eight simulation scenarios
4. Demonstrate clear, structured, and empathetic communication with patients, family members, and team members during simulated encounters
5. Report increased confidence in and readiness for the ABEM Certifying Exam.

### Brief introduction

ABEM recently announced that it will transition to a new format for its oral certification exam. This shift underscores the need for a comprehensive, simulation-based curriculum to prepare EM residents for board certification. This eight-case curriculum also focuses on a wide variety of core EM competencies critical to EM residency training,^2^ including clinical decision-making, task switching, delivering difficult information, conflict resolution, procedural skills, and ultrasound.

### Problem identification, general and targeted needs assessment

In 2026, ABEM will retire its Oral Exam and replace it with the Certifying Exam as part of the testing required for EM physicians to become board-certified.^3^ While ABEM has provided sample case summaries and videos demonstrating each of the eight content areas of the exam on its webpage,^4^ to our knowledge there are no other resources that specifically exist to aid in the preparation for the Certifying Exam. To adequately prepare their trainees for the Certifying Exam, EM residency programs would benefit from a comprehensive simulation-based curriculum that includes an overview of the exam structure, a sample case for each content area, scripts for examiners and standardized participants (SPs), scoring rubrics, debriefing guides, and a list of necessary personnel and equipment.

We selected simulation as the primary educational modality for the curriculum based on its demonstrated effectiveness in EM residency training and its alignment with the goals of the ABEM Certifying Exam. High-fidelity simulation enables deliberate practice of both cognitive and procedural skills, which is essential for preparing residents for high-stakes assessments like the ABEM Certifying Exam.^5^ Additionally, simulation offers a safe, controlled environment where learners can engage in complex, high-acuity scenarios without risk to patients, while receiving structured feedback to guide improvement.^6^ A structured simulation-based curriculum provides flexibility, allowing for both longitudinal skill development in junior residents and high-fidelity mock exam preparation for senior residents, while mirroring the format and expectations of the ABEM Certifying Exam.

This curriculum was developed using a structured, expert-driven approach to ensure educational quality, clinical relevance, and alignment with EM residency training objectives. The final curriculum includes eight simulation cases, each focused on a distinct module corresponding to the new ABEM Certifying Exam. Each case was designed by a team of faculty experts in EM and simulation-based medical education. Those experts were selected based on years of simulation teaching across diverse institutions and active engagement in national simulation collaboratives, including the CORD Simulation Community of Practice. The case development process incorporated principles of instructional design and evidence-based simulation methodology to maximize learner engagement and skill acquisition.

To ensure accuracy, realism, and educational value, each case underwent independent peer review by three or four additional experts. Reviewers applied the Simulation Scenario Evaluation Tool (SSET)^1^ to systematically assess clinical fidelity, scenario flow, and alignment with learning objectives. Revisions were made through an iterative process based on reviewer feedback to enhance clarity, feasibility, and educational impact. Following expert review, the cases were pilot-tested across multiple academic institutions, encompassing a variety of EM residency programs with diverse training environments. Faculty facilitators and resident learners at each site engaged with the simulation scenarios and provided structured feedback via the SSET and a modified usability survey, evaluating case realism, implementation logistics, and learner experience. In addition to institutional pilots, the curriculum was also implemented and evaluated at the Society for Academic Emergency Medicine Annual Meeting in May 2025 (Philadelphia, PA). This broader testing environment allowed for comprehensive feedback on scenario clarity, usability, and relevance to exam preparation. Feedback from these multi-institutional and national pilots informed further revisions to enhance the curriculum’s adaptability, scalability, and readiness for widespread implementation.

### Goals of the curriculum

The goal of this curriculum is to provide a comprehensive, simulation-based case collection designed to prepare EM residents for the new ABEM Certifying Exam. It includes an overview of exam structure, sample cases representing each content area, examiner and standardized patient (SP) scripts, scoring rubrics, debriefing guides, and additional resources to promote consistent, high-quality training. This curriculum serves as a centralized, ready-to-implement resource for EM residency programs, offering peer-reviewed cases that closely replicate the anticipated exam format and reducing the need for programs to develop their own materials.

### Objectives of the curriculum

Upon completion of the curriculum, learners should be able to:

1. List the eight content categories of the ABEM Certifying Exam and describe the overall exam structure
2. summarize the key clinical concepts and examination strategies for each of the eight content categories of the ABEM Certifying Exam
3. apply clinical decision-making, ultrasound, and procedural skills by successfully completing all eight simulation scenarios
4. demonstrate clear, structured, and empathetic communication with patients, family members, and team members during simulated encounters
5. report increased confidence in and readiness for the ABEM Certifying Exam.

### Educational Strategies

All modules are simulation based. Please see the curriculum chart for more details.

### Associated Content

- Appendix A: Curriculum Chart
- Appendix B: Overview of the American Board of Emergency Medicine Certifying Exam

### Results and tips for successful implementation

This curriculum is designed to be delivered as a simulation of the ABEM Certifying Exam, with each curriculum module representing a case type, thus providing residents with a realistic testing experience. Alternatively, individual cases can be implemented to provide targeted practice of specific exam domains based on learner needs. For the most authentic experience, we recommend administering the curriculum in a one-on-one format, allowing learners to engage fully in the simulation and receive personalized, structured feedback. In certain educational contexts, small-group sessions or the use of a senior resident as a mock facilitator can also be effective, particularly for reinforcing group learning or providing insight into examiner perspectives.

To maximize engagement and realism, we recommend conducting the simulations in a setting that closely resembles a typical emergency department (ED) environment. Whenever possible, SPs should be incorporated to portray patient and family member roles, with flexibility to adapt character demographics as needed. When SPs are not available, facilitators can assume character roles, or the cases can be delivered in an oral board format with learners verbalizing their clinical reasoning and planned actions.

Facilitators should be well-versed with both the individual case objectives and the overall structure of the ABEM Certifying Exam (Appendix A) to ensure consistent and effective delivery. Facilitators and SPs should adhere to case scripts to provide learners with a standardized and equitable experience.

For cases involving technical skills (eg, ultrasound, procedures), we recommend incorporating appropriate equipment, including functional ultrasound machines and commercially available task trainers, to enhance realism and allow for accurate assessment of procedural competence. In resource-limited settings, homemade procedural models or oral board-style assessments focused on verbalization of procedural steps can serve as effective alternatives.

In alignment with the ABEM Certifying Exam structure, Clinical Care cases (eg, Clinical Decision-Making, Prioritization) should be allocated 15 minutes, while Communication and Procedural cases (eg, Reassessment, Difficult Conversations, Managing Conflict, Ultrasound, Procedures, Patient-Centered Communications) should be allotted 10 minutes. Each case includes a debriefing guide to support facilitators in conducting structured, reflective debriefing sessions, ensuring educational objectives are reinforced following each scenario.

We received feedback on the overarching curriculum from four reviewers (Table 1), who appreciated the work but requested a less dense chart. A total of 91 people provided feedback on the eight cases (Table 2); 22 were facilitators and 69 were learners. Facilitator feedback was consistently positive across all eight simulation cases (Table 3), with high ratings for the clarity of learning objectives, scenario flow, material integration, ease of use, and overall quality. Facilitators reported confidence in the cases and expressed interest in using them for ABEM Certifying Exam preparation. Resident evaluations reflected these positive findings, with learners rating the cases highly for quality, clarity, and educational value. Residents specifically emphasized the realism of the scenarios and their applicability to clinical decision-making, communication, and procedural skills. Detailed feedback data for each scenario can be found within the individual case materials.

### Evaluation and Feedback

Based on multi-institutional pilot-testing and comprehensive feedback from facilitators and learners, several targeted revisions were made to improve the clarity, consistency, and usability of the curriculum. Examiner scripts were standardized to ensure consistent delivery of prompts and interactions across learners. Similarly, SP scripts were expanded to include detailed, scenario-specific prompts designed to elicit the intended learner responses and support reliable assessment. Critical action checklists were revised to include more specific, observable behaviors and clearer criteria for satisfactory performance while accommodating variations in clinical practice.

To enhance the educational value of the simulation experience, debriefing guides were revised for each case to include structured frameworks and suggested discussion points aligned with learning objectives. Additional guidance was provided for setting up the simulation environment, allowing for flexibility based on institutional resources. Collectively, these changes ensure that the curriculum supports a standardized yet adaptable experience that is both educationally rigorous and practical for diverse EM residency training settings.

## Figures and Tables

**Table:** 

Topic	Recommended Educational Strategy	Educational Content	Objectives	Learners	Timing, Resources Needed	Recommended Assessment, Milestones Addressed
Content Overview	Asynchronous material describing the content and scoring of the ABEM Certifying Exam.	Categorized outline of the ABEM Certifying Exam, including scoring criteria for each content area and links to sample videos.	List the 8 content areas of the ABEM Certifying Exam. Identify key learning points and strategies for each of the 8 exam content areas.	EM physicians preparing for the ABEM Certifying Exam.	Timing: Asynchronous. Examiners: At least 2. Space: Not applicable. Equipment: Handout.	Assessment: Each content area has a scoring checklist. Milestones: Each content area addresses different milestones.
Clinical Decision-Making	Single-station OSCE	Thyroid Storm Review of clinical decision-making, including conducting a targeted history and physical exam, developing a differential diagnosis for tachycardia and altered mental status, recognizing thyrotoxicosis, and initiating appropriate treatment.	Verbalize key pertinent historical and physical exam findings in a young female patient presenting with altered mental status. Formulate a prioritized differential diagnosis based on the history and physical exam. Order appropriate diagnostic studies and recognize abnormalities suggesting thyroid storm. Describe pathophysiology, management, and rationale of sequential pharmacologic therapy in thyroid storm. Communicate patient’s medical care and course to family. Review essential disposition actions including consultations and level of care for admission.	EM physicians preparing for the ABEM Certifying Exam.	Timing: 15 minutes (case), 5 minutes (debriefing). Examiners: 2. Space: Small classroom. Equipment: Table, 3 chairs, computer or printed handouts to show case materials.	Assessment: Scoring checklist. Milestones: PC1, PC3, PC4, PC5, PC6, MK1, ICS1.
Difficult Conversations	Single-station OSCE	Death Notification Framework for delivering a death notification with clarity and compassion, including creating a supportive environment, assessing understanding, responding empathetically, and providing closure.	Establish rapport with the patient’s family by initiating introductions and creating a supportive environment Assess the family’s baseline understanding of the patient’s condition by using open-ended questions and active listening to elicit their perspective. Communicate the patient’s death clearly and compassionately , using concise, non-technical language Demonstrate empathy by responding appropriately to emotional reactions, validating concerns, and addressing questions thoughtfully Provide closure to the conversation by summarizing key points, offering emotional support, and clarifying the next steps in the patient’s care.	EM physicians preparing for the ABEM Certifying Exam.	Timing: 10 minutes (case), 10 minutes (debriefing). Examiners: 1. Space: Simulated ED room Equipment: SP (portraying the patient’s family member), 2 chairs, box of tissues.	Assessment: Scoring checklist. Milestone: ICS1.
Managing Conflict	Single-station OSCE	The Difficult Consultant Strategies for effective conflict management, including introducing and explaining a plan, expressing empathy, validating concerns, recognizing differing viewpoints, fostering shared goals, and reaching resolution.	Review format and become familiar with an ABEM Certifying Exam conflict management communication case. Demonstrate the ability to initiate the consultation call, establish rapport, and present a concise, evidence-based summary of the patient’s STEMI findings. Recognize concerns raised by the cardiologist and respond with an empathetic acknowledgment (eg, validating workload, uncertainty, or resource constraints) to support a collaborative tone. Articulate differing viewpoint by referencing objective clinical data (eg, ST-segment elevations, ongoing chest pain, risk from delays) when conflict is encountered and justify why urgent catheterization lab activation is indicated. Identify shared goals in optimizing patient care (reducing myocardial damage and preventing deterioration) and use these to negotiate a mutually acceptable plan.	EM physicians preparing for the ABEM Certifying Exam.	Timing: 10 minutes (case), 10 minutes (debriefing). Examiners: 1. Space: Simulated ED room. Equipment: SP (portraying the cardiology consultant), table, 2 chairs.	Assessment: Scoring checklist. Milestones: SBP1, SBP3, PROF1, ICS2.
Patient-Centered Communications	Single-station OSCE	Threatened Miscarriage Approach to patient-centered communication, including exploring the patient’s understanding, demonstrating empathy, explaining diagnosis and management, collaborating on a care plan, and sustaining empathy throughout the interaction.	Establish a supportive and compassionate environment through verbal and non-verbal communication when engaging with a patient experiencing distress, anxiety, or grief related to potential pregnancy loss. Actively explore the patient’s understanding, concerns, values, and goals related to their pregnancy and presenting symptoms. Recognize and normalize a range of emotional reactions, offering validation and support regardless of the patient’s obstetric history or desired pregnancy outcomes. Clearly explain the diagnosis of a “threatened miscarriage,” outlining its clinical implications, inherent uncertainty, and potential outcomes. Review the results of any imaging or lab studies succinctly and empathetically. Collaborate with the patient to develop a mutually agreeable care plan, including medical recommendations, appropriate follow-up, monitoring, and return precautions.	EM physicians preparing for the ABEM Certifying Exam.	Timing: 10 minutes (case), 10 minutes (debriefing). Examiners: 1. Space: Simulated ED room. Equipment: SP (portraying the patient), exam table, 2 chairs.	Assessment: Scoring checklist. Milestones: ICS1, PROF1, SBP3, MK1, PROF2, PC4.
Prioritization	Single-station OSCE	Run this board: Septic shock, Acute Coronary Syndrome, Small Bowel Obstruction and Penetrating Chest Trauma Approach to triaging and managing 8 patients of varying acuity presented through staged tracking boards, with evolving data, competing priorities, and limited team resources.	Become familiar with format of a prioritization case (a component of the ABEM Certifying Exam). Practice their ability to prioritize multiple patients and provide stabilizing care. Consider changes in status/patient acuity/new cases as presented. Understand how to utilize team resources appropriately.	EM physicians preparing for the ABEM Certifying Exam.	Timing: 15 minutes (case), 15 minutes (debriefing). Examiners: 2. Space: Small classroom. Equipment: Table, 3 chairs, computer or printed handouts to show case materials.	Assessment: Scoring checklist. Milestones: PC2, PC3, ICS2, SBP1.
Procedures	Single-station OSCE	Procedure Case: Neonatal LP Review of neonatal LP, including obtaining Informed Consent using patient-centered language, preparing for and performing the procedure, and managing providing post-procedure care including interpretation of cerebrospinal fluid (CSF) results.	Describe the indications and contraindications associated with performing a neonatal lumbar puncture (LP). Obtain informed consent for a neonatal LP, using clear, patient-centered language to explain the procedure and to discuss risks, benefits, and alternative options. Demonstrate proper preparation for a neonatal LP, including equipment setup, patient positioning, patient monitoring, use of sterile technique, and analgesia. Perform a neonatal LP on a procedural task trainer with technical proficiency, demonstrating proper needle insertion, cerebrospinal fluid (CSF) collection, and adherence to sterile technique. Outline appropriate post-procedure management for the patient, including interpreting CSF results, initiating appropriate treatment, monitoring for complications, and providing caregivers with clear follow-up guidance.	EM physicians preparing for the ABEM Certifying Exam.	Timing: 10 minutes (case), 10 minutes (debriefing). Examiners: 1. Space: Small classroom. Equipment: Neonatal LP task trainer, antiseptic solution, sterile gloves, sterile drapes, local anesthetic, LP needles, CSF collection tubes, manometer, computer or printed handouts to show case materials.	Assessment: Scoring checklist. Milestones: PC8, MK2, PBLI1, ICS1.
Reassessment/Trouble-shooting	Single-station OSCE	Reassessment: Pulmonary Edema Approach to reassessment, including identifying signs of clinical deterioration and applying effective troubleshooting strategies to manage acute decompensation.	Demonstrate competency with the new ABEM Certifying Exam Reassessment case format. Demonstrate the ability to evaluate new information and integrate it into an existing care plan. Recognize signs and symptoms of pulmonary edema. Review possible etiologies of acute respiratory distress and the evaluation/work up to differentiate and diagnose those causes.6. Manage pulmonary edema (PE) including implementing afterload reduction, positive pressure ventilation, and diuresis.	EM physicians preparing for the ABEM Certifying Exam.	Timing: 10 minutes (case), 10 minutes (debriefing). Examiners: 1. Space: Simulated ED room. Equipment: SP (portraying the patient), exam table, 2 chairs.	Assessment: Scoring checklist Milestones: PC2, MK1, PC3, PC4, SBP2, PC5, ICS1.
Ultra-sound	Single-station OSCE	Pulmonary Embolism US Case Review of the use of focused cardiac and vascular ultrasound, including acquiring high-quality diagnostic images, accurately interpreting findings, and preparing for and performing ultrasound-guided central venous access.	Obtain and interpret the parasternal short-axis view of the heart to assess right ventricular size and function. Identify ultrasound findings suggestive of PE on cardiac short-axis view, including right ventricular dilation and septal bowing. Demonstrate appropriate probe selection and positioning to obtain optimal images of the heart and interior vena cava (IVC). Evaluate the IVC using a subxiphoid or longitudinal view to assess distension and lack of respiratory collapse as supportive findings for elevated right heart pressures. Identify the anatomy of the neck vasculature, differentiate between the internal jugular vein and carotid artery, and select the appropriate puncture site. Perform ultrasound-guided central venous catheterization via the right internal jugular vein, using sterile technique and real-time guidance.	EM physicians preparing for the ABEM Certifying Exam.	Timing: 10 minutes (case), 10 minutes (debriefing). Examiners: 1. Space: Small classroom or simulated ED room. Equipment: SP (serving as the ultrasound model), exam table, ultrasound machine, computer or printed handouts to show case materials, ultrasound-capable internal jugular CVC insertion task trainer, CVC insertion kit.	Assessment: Scoring checklist. Milestone: PC8.

ABEM, American Board of Emergency Medicine; CSF, cerebrospinal fluid; CVC, central venous catheter; ED, emergency department; EM, emergency medicine; ICS, interpersonal and communication skills; LP, lumbar puncture; MK, medical knowledge; OSCE, objective structured clinical examination; PBLI, practice-based learning and improvement; PC, patient care; PROF, professionalism; SBP, systems-based practice; SP, standardized patient.

**Figure:**
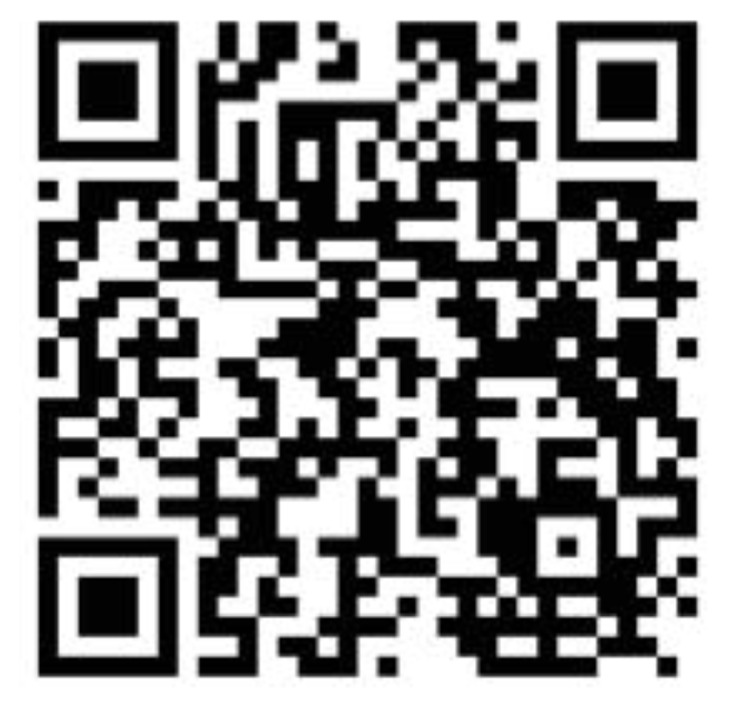
Watch Example

**Figure:**
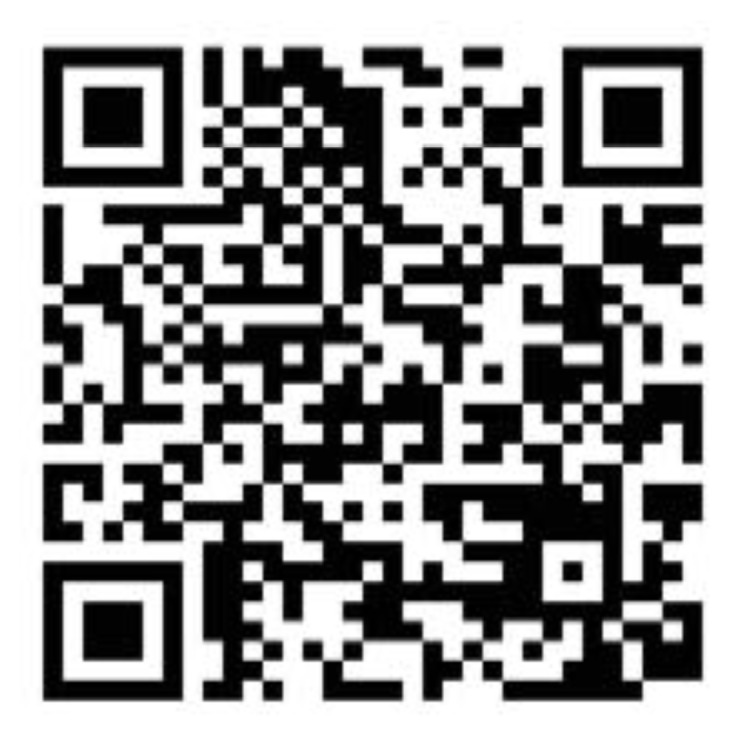
Watch Example

**Figure:**
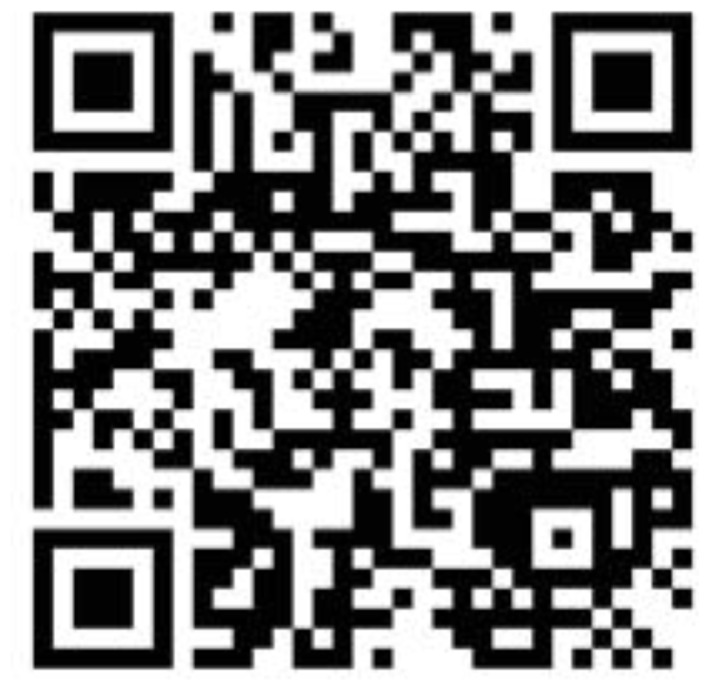
Watch Example

**Figure:**
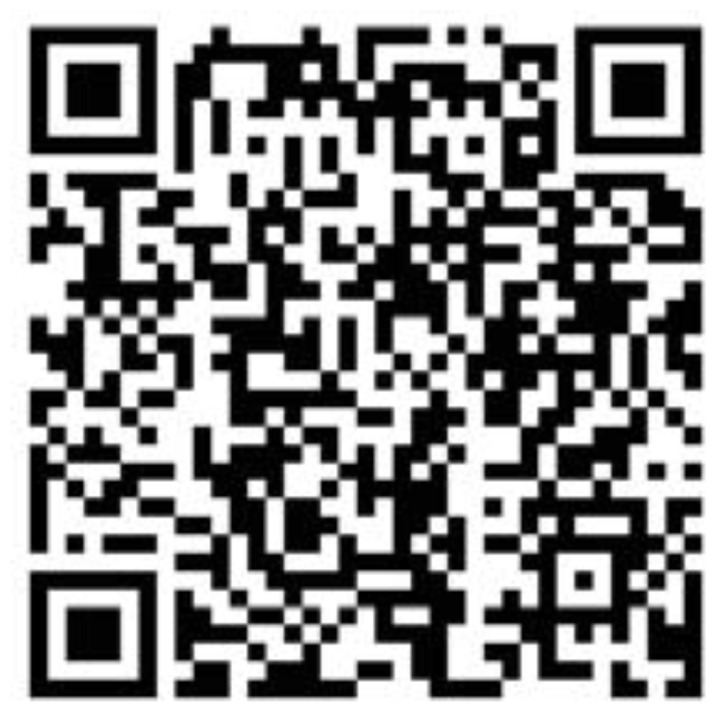
List of Procedures

**Figure:**
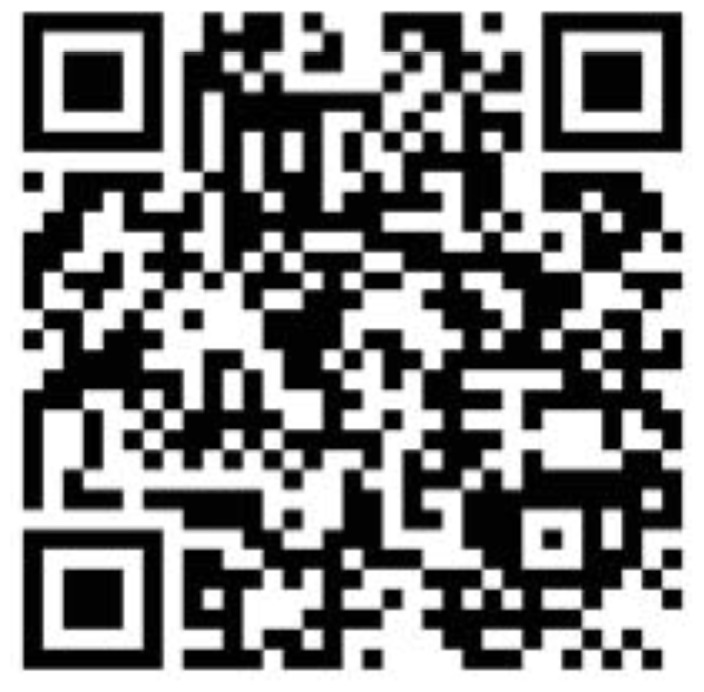
Watch Example

**Figure:**
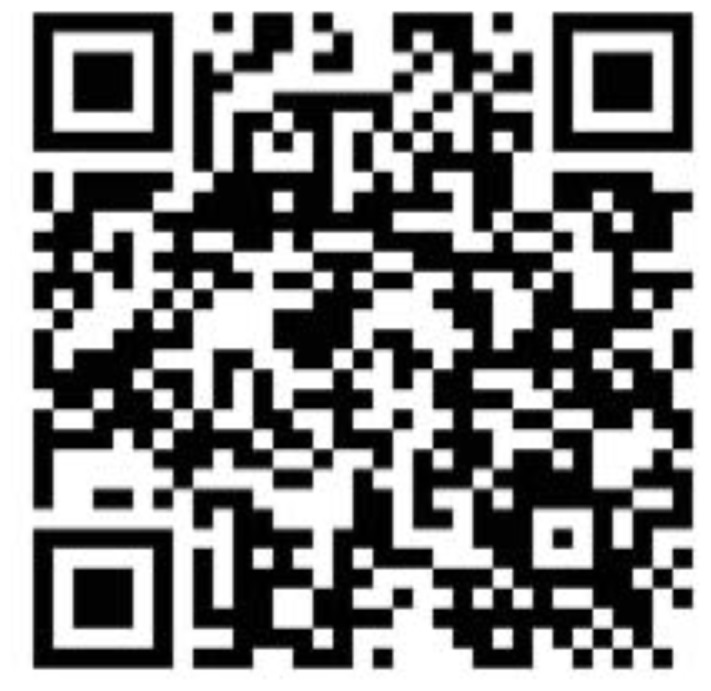
Watch Example

**Figure:**
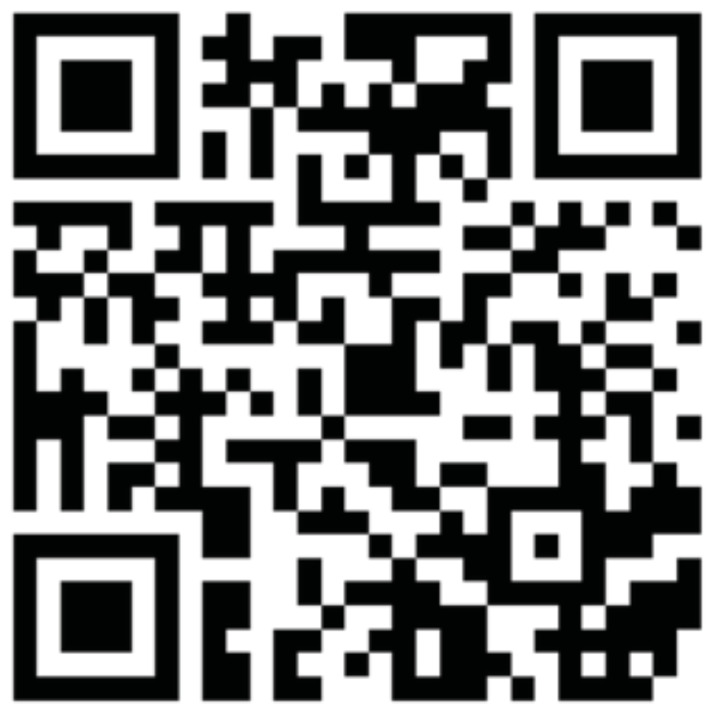
Watch Example

**Figure:**
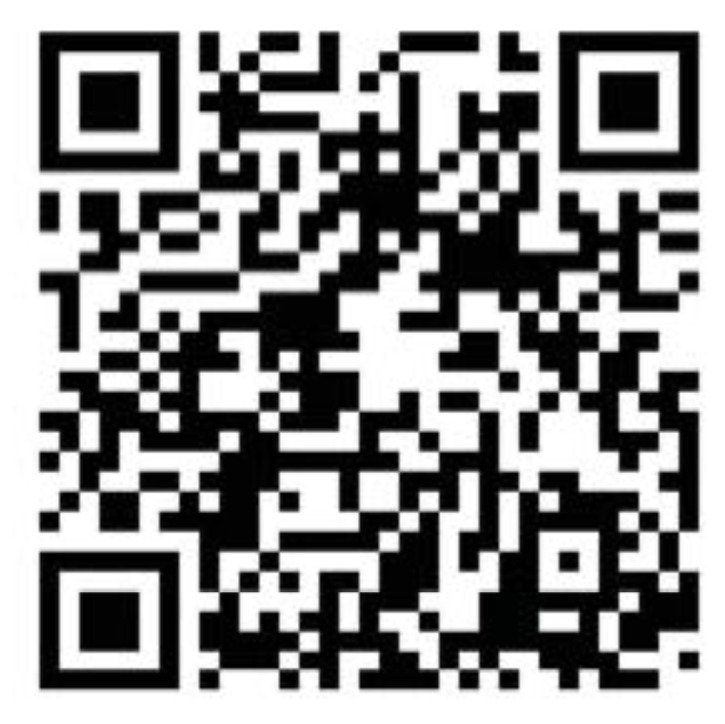
Watch Example

**Figure:**
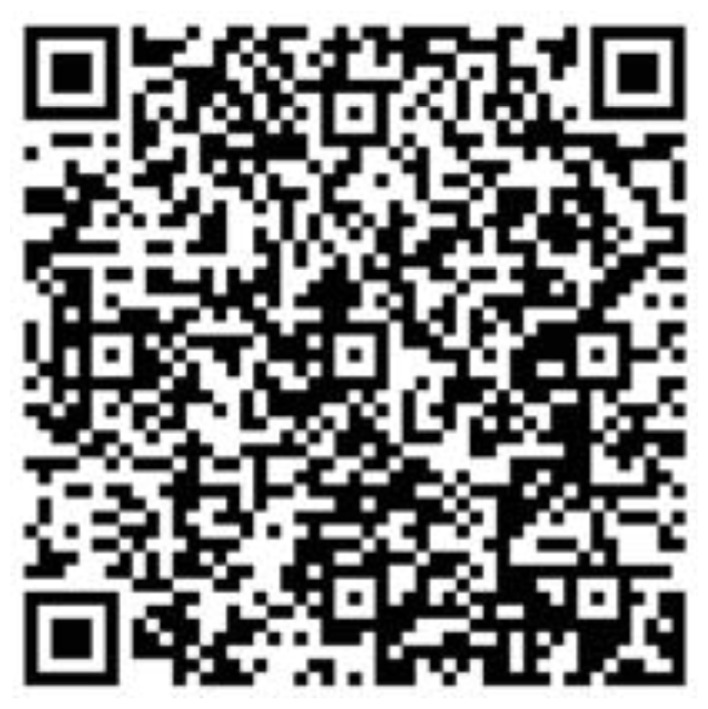
List of Ultrasounds

**Figure:**
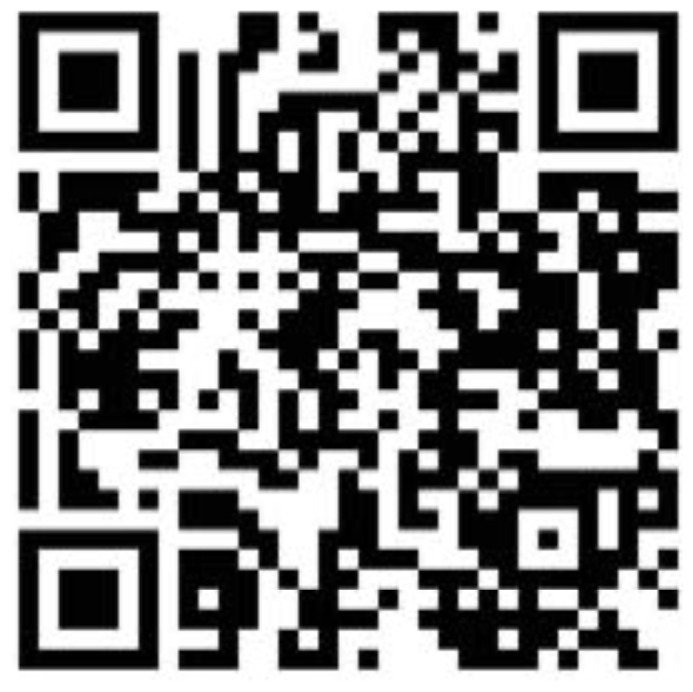
Watch Example

**Figure:**
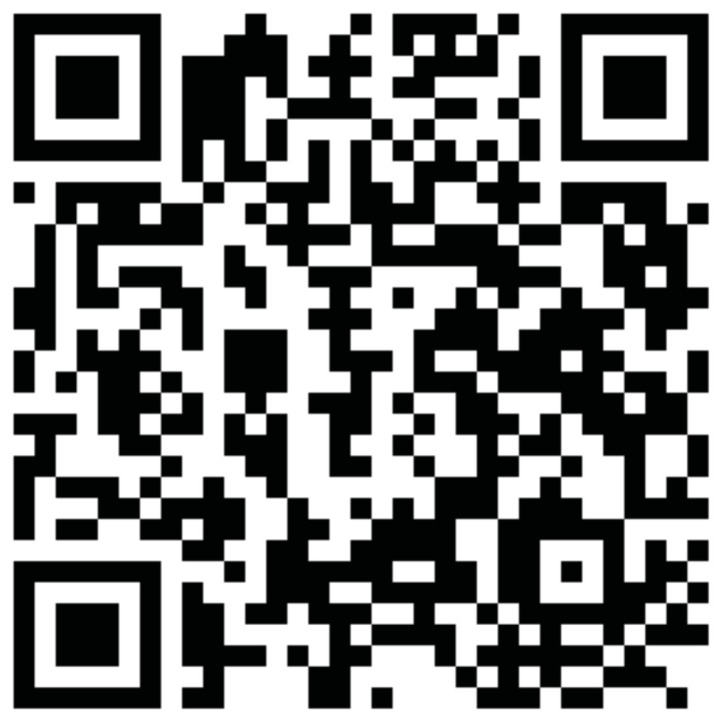
Good luck!

**Table 1:** Curriculum feedback

Question	Mean (n=4)
I would like to use this curriculum for ABEM certifying exam practice	4.3
I thought the curriculum was easy to use	3.8
I found the materials in this curriculum were well integrated	4.0
I would imagine that most people would learn to use this curriculum very quickly	4.0
I felt very confident using the curriculum for ABEM certifying exam practice	3.8

Scored on a Likert scale (1 = strongly disagree, 5 = strongly agree)

**Table 2:** Total number of facilitators and learners per case.

Case	Expert and Facilitator Reviewers	Learners / Mock Examinees	Total Participants
Clinical Decision Making	5	1	6
Difficult Conversation	5	19	24
Managing Conflict	5	16	21
Patient-Centered Communication	3	5	8
Prioritization	2	8	10
Procedure	3	6	9
Reassessment	4	11	15
Ultrasound	1	4	5

**Table 3:** Mean facilitator and learner usability scores.

Case	Mean Facilitator Usability Score	Mean Learner Usability Score	Notes
Clinical Decision Making	4.3	4.7	Strong exam-prep value
Difficult Conversation	4.8	4.9	Very high learner clarity and educational value
Managing Conflict	5	4.8	Learners requested clarification of STEMI transfer options
Patient-Centered Communication	5	4.7	Strong clarity and usability
Prioritization	n/a	4.3	High educational value, clarity moderate
Procedure	4	4.6	Very strong clarity and relevance
Reassessment	5	4.7	Good pacing, clear materials
Ultrasound	n/a	4.4	Clear materials, high realism

Scored on a Likert scale (1 = strongly disagree, 5 = strongly agree)

## DIDACTICS AND HANDS-ON CURRICULUM

### Overview of the American Board of Emergency Medicine Certifying Exam

This handout summarizes the case types included in the American Board of Emergency Medicine Certifying Exam and provides QR codes linking to sample videos and other resources.

**Exam Structure:**

Two assessment types: **Clinical Care** cases and **Communication and Procedural** cases

**Clinical Care** cases:

- Content areas:
  - Clinical Decision-Making
  - Prioritization
- 4 cases total per exam session
- For each case, candidates will have 5 minutes to review the provided task sheet prior to entering the room for a **15-minute case scenario**
- There will be 2 examiners in each room

**Communication and Procedural** cases:

- Content areas:
  - Reassessment/troubleshooting (Communication)
  - Difficult conversations (Communication)
  - Managing conflict (Communication)
  - Patient-centered communications (Communication)
  - Ultrasound (Procedural)
  - Procedures (Procedural)
- 6 cases total per exam session
- For each case, candidates will have 5 minutes to review the provided task sheet prior to entering the room for a **10-minute case scenario**
- Communication case types:
  - There will be a standardized patient actor in each room
- Procedural case types:
  - There will be an examiner (+/− standardized patient actor) in each room
  - Procedure cases: will use relevant simulation equipment depending on scenario
  - Ultrasound cases: candidates will operate ultrasound probe; examiner will operate ultrasound machine and adjust machine settings (eg, depth, gain, mode, transducer) based on candidate instructions

#### Clinical Decision-Making

Assesses your diagnostic and management skills in an undifferentiated patient scenario.

Key Skills:

- Perform focused history and physical exam
- Formulate differential diagnosis
- Perform diagnostic testing and interpretation
- Implement stabilization and treatment
- Reassess and provide anticipatory guidance
- Discuss pathophysiology

#### Prioritization

Assesses your ability to manage multiple patients and triage effectively.

Key Skills:

- Stratify patient acuity
- Provide immediate stabilizing care
- Triage evolving or new cases
- Coordinate team resources

#### Difficult Conversations

Assesses your ability to disclose sensitive, unwanted, or unexpected information to a patient or family member.

Key Skills:

- Establish rapport
- Determine baseline knowledge
- Disclose information
- Respond and react appropriately
- Provide closure

#### Procedures

Assesses your understanding and technical performance of emergency procedures.

Key Skills:

- Describe indications and contra-indications
- Prepare for procedure
- Demonstrate technical proficiency
- Provide post-procedure care

#### Patient-Centered Communications

Assesses your ability to effectively communicate and deliver empathetic, patient-focused care.

Key Skills:

- Establish rapport and respect
- Facilitate mutual understanding
- Share results
- Engage in shared decision-making

#### Reassessment

Assesses your response to new or changing information during a case.

Key Skills:

- Integrate new info
- Modify treatment
- Anticipate next steps
- Consider systems-based factors

#### Ultrasound

Assesses ultrasound image acquisition, interpretation, and clinical application.

Key Skills:

- Obtain clear ultrasound images
- Interpret images accurately
- Provide patient-centered care

**More Information:** For full details, visit the official ABEM website:
